# Ni-Doped SnO_2_ Gas Sensor Array Enabled High-Randomness PUF for Hardware Security Applications

**DOI:** 10.3390/mi17050597

**Published:** 2026-05-14

**Authors:** Zexin Ji, Xiaowei Zhang, Zhanbo Chen, Shanshan Wang, Wenbo Zhang, Hao Ye, Xiangyu Li

**Affiliations:** 1 Faculty of Electrical Engineering and Computer Science, Ningbo University, Ningbo 315211, China; 2311100221@nbu.edu.cn (Z.J.); zhangxiaowei@nbu.edu.cn (X.Z.); 2411100004@nbu.edu.cn (Z.C.); 2 Qian Xuesen Collaborative Research Center of Astrochemistry and Space Life Sciences, Ningbo University, Ningbo 315211, China; 3 Institute of Drug Discovery Technology, Ningbo University, Ningbo 315211, China; 4 School of Astronautics, Harbin Institute of Technology, Harbin 150001, China; zwb0311@hit.edu.cn; 5 College of Electrical and Electronic Engineering, Wenzhou University, Wenzhou 325035, China; 6 Ningbo Institute of Digital Twin, Eastern Institute of Technology, Ningbo 315042, China; lixiangyu@nbu.edu.cn; 7 Ningbo Key Laboratory of Spatial Intelligence and Digital Derivative, Ningbo 315200, China

**Keywords:** gas sensor PUF (GS-PUF), Ni-doped SnO_2_ nano-sensor array, random resistance balancing algorithm, physical unclonable function (PUF)

## Abstract

With the growing security requirements of sensor nodes in Internet of Things (IoT) systems, conventional silicon-circuit-based physical unclonable functions (PUFs) still face limitations in circuit overhead, design complexity, and system integration. To address these challenges, this paper proposes a lightweight gas sensor PUF (GS-PUF) design based on a Ni-doped SnO_2_ nanoscale gas sensor array. The proposed method exploits both the unavoidable process randomness introduced during sensor fabrication and the device-to-device electrical response variations induced by gas–material interactions as entropy sources, thereby enabling high-quality PUF response generation. At the device level, Ni-SnO_2_ nanomaterials are prepared by electrostatic spray deposition (ESD), and an indirectly heated gas sensor array is constructed to enhance the sensitivity and stability of the sensing response. At the algorithmic level, a random resistance balancing algorithm based on multi-sensor combinational comparison is proposed. By randomly comparing the summed resistances of multiple sensor clusters, a 128-bit multi-bit PUF response is generated, while the uniformity and independence of the output bits are effectively improved. Experimental results demonstrate that the proposed GS-PUF exhibits excellent randomness, uniqueness, and reliability: the information entropy of the PUF responses is greater than 0.99, approaching the ideal value; the probabilities of output bits “1” and “0” are 0.4988 and 0.5012, respectively, indicating a well-balanced distribution; the inter-device uniqueness reaches 49.8%, close to the ideal value of 50%; all items in the NIST randomness test suite are passed, with all *p*-values exceeding 0.01 and the minimum *p*-value being 0.0368, confirming a high level of statistical randomness confidence. In addition, long-term measurements under fixed laboratory conditions show that the PUF response reliability remains above 96%. Compared with other sensor-based PUFs, the proposed method provides a lightweight sensing-security integration approach for IoT sensor nodes by reusing intrinsic gas-sensor response variations and avoiding an additional dedicated silicon PUF circuit.

## 1. Introduction

With the rapid development of Internet of Things (IoT) technologies, sensor nodes have been widely deployed in application scenarios such as environmental monitoring, industrial control, and intelligent manufacturing [1,2,3,4,5,6,7]. As critical interfaces between the physical world and information systems, sensors not only perform data acquisition but also face severe security threats. However, because IoT devices are typically resource-constrained, geographically distributed, and operated for long periods in unattended environments, conventional cryptography-based security mechanisms struggle to simultaneously satisfy the requirements of cost, power consumption, and security. Therefore, the exploration of lightweight and highly secure hardware security primitives has become an important research focus [8,9,10,11].

Physical unclonable functions (PUFs) utilize the unavoidable random process variations introduced during device fabrication as entropy sources to generate unique and difficult-to-reproduce responses, and have shown considerable potential in device authentication and key generation [12,13,14,15,16]. Conventional silicon-based PUFs generally require additional circuit structures, resulting in high design complexity and substantial hardware overhead [17,18,19]. In recent years, PUF designs based on the intrinsic characteristics of sensors have attracted increasing attention. By directly exploiting the randomness embedded in sensor output signals, such designs can provide security functions and are expected to enable a lightweight security architecture integrating sensing, storage, computing, and protection.

Among various sensor types, semiconductor gas sensors are widely used in gas detection and environmental perception because of their simple structure, high sensitivity, and ease of integration [20,21]. Nevertheless, existing gas-sensor-based PUF designs still suffer from limitations in randomness, uniqueness, and response stability, which restrict their deployment in high-security applications [22,23,24,25]. To overcome these limitations, this paper proposes a highly sensitive gas sensor PUF design based on Ni-doped SnO_2_ nanomaterials. By introducing Ni doping to regulate the interfacial properties of the sensing material, the gas response characteristics are enhanced. Combined with a multi-sensor array and a random resistance balancing algorithm, high-quality multi-bit PUF responses are generated. Experimental results indicate that the proposed method significantly improves PUF randomness and uniqueness while maintaining enhanced sensing performance, thereby providing an effective route for low-cost and high-security sensor PUF designs targeting IoT applications.

- Motivation

Although PUFs hold significant promise for hardware security, conventional silicon-based PUFs typically rely on additional circuitry and therefore suffer from large area and power overheads as well as high design complexity, making them unsuitable for resource-constrained IoT devices [26,27,28,29]. Meanwhile, as core components of the perception layer, sensor nodes often lack intrinsic security mechanisms and are vulnerable to attacks, creating an urgent need for lightweight security solutions. Sensor-intrinsic PUFs offer a new pathway toward integrating sensing and security; however, they still face challenges such as insufficient response stability, limited information entropy, and imbalanced bit distributions. To address these challenges, this work introduces Ni-doped SnO_2_ nanomaterials to improve sensing performance and combines a multi-sensor array with a random resistance balancing algorithm to generate high-quality multi-bit PUF responses with enhanced randomness and uniqueness.

B. Related Work

Conventional PUF designs are mostly implemented by exploiting fabrication-induced variations in silicon devices, such as arbiter PUFs and SRAM PUFs. These designs generate unique responses by extracting differences in circuit delay or storage states and have been widely applied in device authentication and key generation. However, such approaches generally rely on dedicated circuit structures, which introduce additional hardware overhead and increase design complexity. To reduce system cost and improve integration, researchers have begun to investigate PUF designs based on the intrinsic properties of sensors. Willers et al. quantized the analog output signals of MEMS gyroscopes and successfully generated high-entropy keys [30]. Labrado proposed a PUF scheme based on the mean output voltage of a piezoelectric sensor, although it strongly depends on AC excitation and exhibits limited noise immunity [31]. Degada et al. further exploited the uncertainty of photoresistors to implement a true random number generator (TRNG), demonstrating the potential of intrinsic sensor randomness [32]. Previous work has also attempted to construct PUFs based on magnetic sensor arrays by using device process variations as entropy sources to realize security mechanisms without additional hardware [33]. Although these studies validate the feasibility of sensor PUFs, further improvements are still required in response stability, randomness, and multi-bit generation capability.

## 2. Gas Sensor

### 2.1. Sensor Fabrication

**Figure 1:**
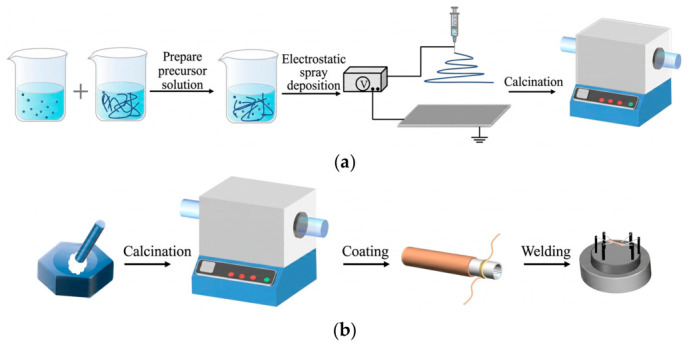
(**a**) Preparation process of oxide nanomaterials; (**b**) Fabrication process of the semiconductor gas sensor.

In this work, Ni-doped SnO_2_ gas-sensitive nanomaterials were prepared by electrostatic spray deposition (ESD), and semiconductor gas sensors were fabricated using an indirectly heated structure. First, SnCl_4_·5H_2_O (525 mg) and polyvinylpyrrolidone (PVP, 1200 mg) were dissolved in a mixed solvent of N,N-dimethylformamide (DMF, 5 mL) and absolute ethanol (5 mL), followed by magnetic stirring for 5 h to obtain a homogeneous and transparent precursor solution. NiCl_2_·2H_2_O (17 mg) was then added into the SnO_2_ precursor solution as the Ni dopant source, and the mixed solution was further stirred for 2 h to ensure complete dissolution and uniform dispersion of Ni species. This Ni doping condition was used throughout this work for the fabrication of Ni-SnO_2_ gas sensors. Subsequently, the precursor solution was loaded into a syringe, and a high-voltage electric field of approximately 16 kV was applied between the needle and the collector, with the spinning distance controlled at 10–15 cm. Under the electric-field force, fibers were formed and deposited on the collector surface. The deposited film was then placed in a muffle furnace, heated to 600 °C in air, and maintained for 2 h to complete the calcination process, ultimately yielding Ni-doped SnO_2_ nanoparticles. The fabrication processes of the oxide nanomaterials and semiconductor gas sensors are shown in Figure 1.

**Figure 2:**
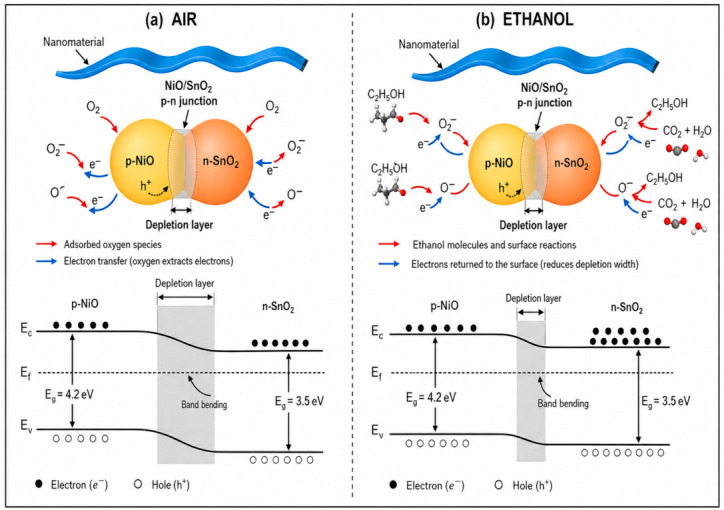
Schematic gas-sensing mechanism and energy-band structure of the Ni-SnO_2_ sensor: (**a**) in air; (**b**) in ethanol.

When NiO is introduced into SnO_2_, a p-n junction is formed at the interface between NiO and SnO_2_ particles. Figure 2 presents a simplified model of the Ni-SnO_2_ sensor exposed to air and ethanol. In this case, because of the large carrier concentration gradient between the two semiconductors, electrons in SnO_2_ and holes in NiO diffuse in opposite directions until the Fermi levels across the p-n junction become aligned. This process establishes a depletion layer at the NiO/SnO_2_ particle interface, which plays a crucial role in improving the sensing performance of the Ni-SnO_2_ sensor. In the Ni-SnO_2_ gas sensor, electrons act as the majority carriers. During oxygen adsorption, free electrons are captured by oxygen species, resulting in an increased depletion-layer width at the p-n junction, as shown in Figure 2a. Once the sensor is exposed to ethanol vapor, surface oxides promote the oxidation of ethanol molecules into CO_2_ and H_2_O, and the captured electrons are released back to the sensor, thereby reducing its resistance. In addition, C_2_H_5_OH releases electrons into NiO; the ensuing electron-hole recombination decreases the hole concentration and increases the electron concentration in NiO. Consequently, the carrier concentration gradient across the two sides of the p-n junction decreases, carrier diffusion is substantially suppressed, and the depletion layer at the interface becomes thinner, as shown in Figure 2b. The presence of p-n junctions in the nanoparticles can markedly enhance the adsorption and decomposition rates of oxygen molecules and ethanol on the sensor surface, enabling excellent sensing performance, including high sensitivity, good repeatability, and shorter response and recovery times. These improvements not only facilitate faster extraction of the required physical feature information but also make such information more stable and reliable, thereby further improving the performance of PUF data generation. Ethanol was selected as the stimulus gas in this work for the following reasons. First, ethanol is a representative reducing gas for SnO_2_-based metal-oxide gas sensors, and its surface reaction with adsorbed oxygen species can release trapped electrons back to the sensing material, resulting in a clear resistance decrease. Second, the NiO/SnO_2_ p-n junction can enhance the adsorption and decomposition of oxygen and ethanol molecules on the sensor surface, leading to a stronger and more repeatable resistance response. Such stable resistance modulation is beneficial for extracting device-to-device response deviations as PUF entropy sources. Therefore, ethanol was used as a proof-of-concept stimulus to validate the feasibility of the proposed Ni-SnO_2_-based GS-PUF design.

For device construction, an indirectly heated semiconductor gas sensor structure was adopted. The prepared Ni-SnO_2_ nanomaterials were mixed with deionized water at a selected ratio to form a slurry, which was uniformly coated onto the surface of an alumina tube equipped with comb-shaped gold electrodes. Platinum electrodes were pre-embedded at both ends of the alumina tube for signal extraction, while an alloy heating wire was inserted into the tube to provide a stable operating temperature. The coated device was annealed again at 600 °C for 2 h to improve material adhesion and structural stability. Finally, the heating and measurement electrodes were welded to the sensor base, completing the fabrication of the gas sensor device. This fabrication method yields Ni-SnO_2_ nanomaterials with uniform structures and favorable interfacial characteristics, effectively enhancing the response sensitivity and stability of the sensor toward target gases and providing a reliable physical basis for subsequent PUF feature extraction. To clarify fabrication reproducibility, all Ni-SnO_2_ sensors in this work were prepared using the same precursor composition, stirring procedure, deposition voltage, collection distance, calcination temperature, and annealing duration. Therefore, the fabrication process is repeatable at the process-parameter level. It should be noted that the unavoidable microscopic variations introduced during material deposition, nanoparticle formation, grain distribution, and interfacial-state formation are not regarded as fabrication failures; instead, they provide the physical entropy sources required for GS-PUF response generation. Thus, the proposed GS-PUF relies on controlled process conditions while exploiting unavoidable device-to-device variations for unclonable response extraction.

### 2.2. Characterization of Gas-Sensitive Materials

**Figure 3:**
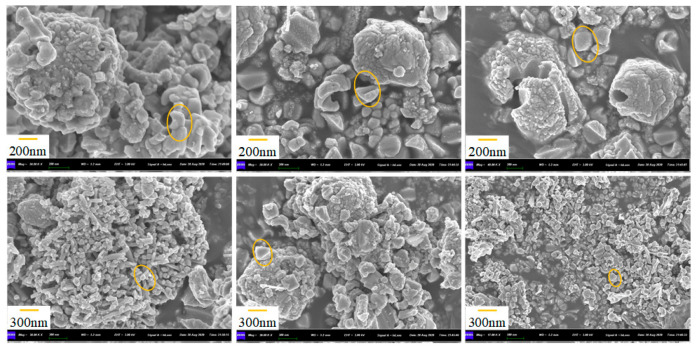
SEM image of Ni-doped SnO_2_ nanoparticles. The orange circles indicate the oxide particle regions.

The SEM characterization of the Ni-SnO_2_ nanoparticles is shown in Figure 3. It can be observed that the Ni-doped oxide powder exhibits a morphology similar to that of pristine SnO_2_ oxide, with the surface composed of irregularly shaped, angular nanoparticles.

**Figure 4:**
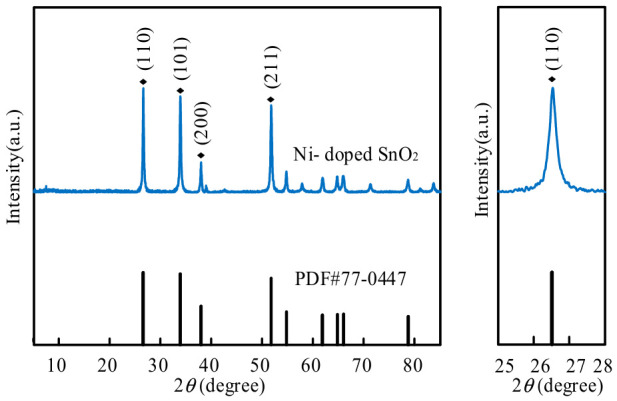
XRD characterization.

The XRD pattern of the nanomaterial sample after high-temperature sintering at 600 °C is shown in Figure 4. Diffraction peaks corresponding to the (110), (101), (200), and (211) planes are observed, matching the standard JCPDS card of SnO_2_ (PDF#77-0447). Because the NiO fraction is very small, Ni doping does not alter the crystal structure of SnO_2_, and no characteristic NiO peaks are observed.

### 2.3. Deviation Characteristics

**Figure 5:**
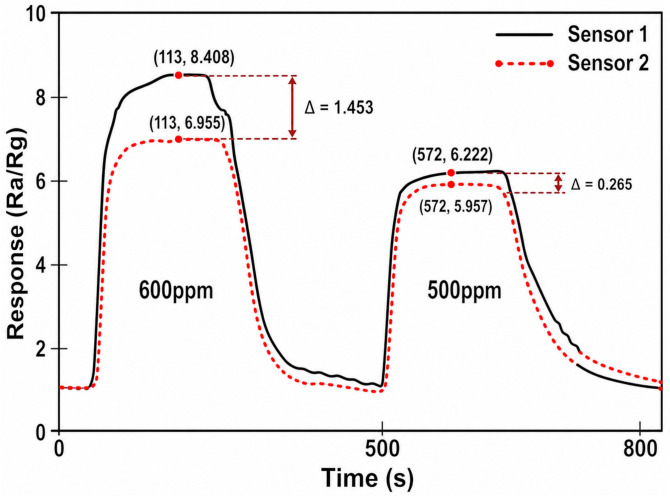
Analysis of sensor response deviation.

The deviation characteristic describes the response differences among different sensors under the same gas stimulus as shown in Figure 5. The fabricated Ni-SnO_2_ gas sensors were tested under ethanol concentrations of 600 ppm and 500 ppm. In the relatively stable stage of the response curves, Sensor 1 and Sensor 2 exhibit evident deviation characteristics. Compared with the SnO_2_ gas sensor, whose signal requires 48 s to reach the stable stage, the Ni-SnO_2_ gas sensor requires only 41 s, representing a 14.6% reduction in stabilization time. This reduction in stabilization time indicates that the adopted Ni doping condition improves the gas-sensing dynamics of SnO_2_, enabling faster extraction of stable resistance features and reducing the waiting time required for PUF response generation.

## 3. PUF Response Extraction

**Figure 6:**
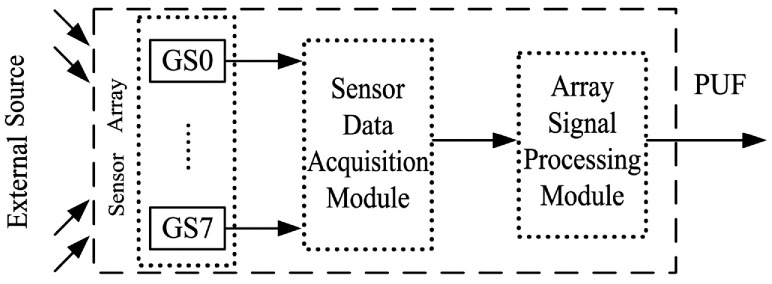
Framework of multi-bit output GS-PUF design.

Because semiconductor gas sensors are inevitably affected by process variations during fabrication, such as nonuniform material deposition, random microstructural defects, and differences in interfacial states, different devices exhibit intrinsic electrical response differences under identical external stimuli. These unclonable features introduced by manufacturing randomness provide a natural entropy-source basis for constructing gas-sensor-based PUFs. In this work, a gas sensor array is used to extract GS-PUF responses, as shown in Figure 6. The overall framework consists of an external stimulus source, a gas sensor array, a data acquisition module, and an array signal-processing module. Specifically, under a unified ethanol environment, which was selected as a representative reducing-gas stimulus for Ni-SnO_2_ sensors, multiple gas sensors in the array operate simultaneously and output their corresponding resistance values.

To generate high-quality, multi-bit, and statistically balanced PUF responses, this work proposes a multi-bit extraction method based on random resistance combinational comparison. The method first selects a fixed number of sensors, such as eight, from the sensor array as basic units and constructs multiple sensor clusters through combinational operations. Specifically, three sensors are selected from the eight sensors each time to form a subset, namely a sensor cluster. The summed resistance values of different clusters are then compared to generate a single-bit output: the output is “1” if the resistance sum of one sensor group is greater than that of the other group, and “0” otherwise. Compared with conventional pairwise comparison methods, this multi-element combinational comparison strategy significantly increases the number of available challenge-response pairs (CRPs). For eight sensors, selecting three sensors to form a subset yields 56 possible combinations, and pairwise comparisons between different subsets can create a combinational space far larger than that of simple comparison, thereby increasing response-generation diversity and modeling complexity. and security. In addition, the summation of multiple sensor responses can average out, to some extent, the noise fluctuation of individual devices and improve overall system stability.

To prevent PUF responses from being biased toward a specific sensor or local feature, a random resistance balancing algorithm is designed in this work as shown in Table 1. By dynamically adjusting the sensor indices involved in comparison, the algorithm enables different sensors to participate in multiple comparison processes in a balanced manner, thereby ensuring favorable randomness and uniform distribution in the output bitstream. In the implementation, the resistance values of eight sensors are used to form an input vector. Two sensor clusters, denoted as the left and right clusters, are generated through cyclic shifting and predefined index combinations, and their resistance sums are compared. Each comparison generates one output bit. Repeating this process eight times produces an 8-bit sub-response, and further iterations, such as 16 rounds, yield a 128-bit PUF response sequence.

Based on the proposed GS-PUF extraction method, its randomness and security can be modeled and analyzed from an information-theoretic perspective. Let the PUF output be a binary response sequence of length N, denoted as R. The single-bit information entropy can be expressed as:

(1) $$H\left(X\right)=-p{log}_{2}p-\left(1-p\right){log}_{2}\left(1-p\right)$$

Here $p=Pr\;(r_{i}=1)$, when the probability of outputting “1” approaches 0.5, the single-bit entropy approaches its maximum value of 1 bit, ensuring high information uncertainty of the overall response. Furthermore, under ideal independent and identically distributed conditions, the total sequence entropy approaches N bits. Meanwhile, the uniformity of the PUF output can be characterized by the proportion of “1” bits, whose ideal value is 0.5. The proposed random resistance balancing algorithm, through multi-sensor combinational rotation and index offsetting, effectively suppresses bias caused by the dominance of individual devices, making the output bitstream statistically closer to a uniform distribution. To evaluate the independence among different response bits, mutual information is introduced as a metric and is defined as:

(2) $$I(X;Y)=\sum_{x\in X}\sum_{y\in Y}p(x,y){log}_{2}\frac{p(x,y)}{p(x)p(y)}$$

When the mutual information approaches zero, different output bits can be regarded as approximately independent. In the proposed method, each bit is derived from different sensor combinations and dynamic index mappings; therefore, the statistical correlation among bits is expected to be reduced by using different sensor combinations and dynamic index mappings. However, this analysis only suggests increased response-generation diversity and should not be interpreted as quantitative evidence of resistance to machine-learning-based modeling attacks. In addition, from the perspective of combinational complexity, selecting three sensors from an array of eight sensors forms C_(8,3)_ = 56 possible sensor clusters. If two different clusters are compared to generate one response bit, the number of possible unordered cluster-comparison pairs is C_(56,2)_ = 1540. In contrast, a conventional pairwise sensor-comparison scheme based on eight sensors only provides C_(8,2)_ = 28 comparison pairs. Therefore, the proposed multi-sensor combinational strategy enlarges the comparison space by approximately 55 times compared with direct pairwise comparison, which helps reduce the dominance of individual sensors and improves response-generation diversity. Compared with conventional pairwise-comparison-based PUF extraction methods, the proposed multi-sensor summation comparison mechanism provides a certain degree of statistical averaging, which can help reduce the influence of noise fluctuations from individual sensors and improve response stability. Moreover, the randomness introduced by the microstructure of gas-sensitive materials and gas–material interactions provides a physical entropy source for GS-PUF response generation. Therefore, the proposed method exhibits favorable statistical characteristics in terms of entropy, uniformity, and uniqueness. It should be noted that the present work mainly evaluates the proposed GS-PUF using statistical metrics, including information entropy, bit uniformity, uniqueness, NIST randomness tests, and reliability. Although the multi-sensor combinational comparison strategy may help reduce response correlation and increase modeling complexity, a quantitative machine-learning-based modeling attack evaluation was not conducted in the current study. Therefore, the modeling-attack resistance of the proposed GS-PUF should be further investigated in future work using representative machine-learning models.

## 4. Test Results and Analysis

**Figure 7:**
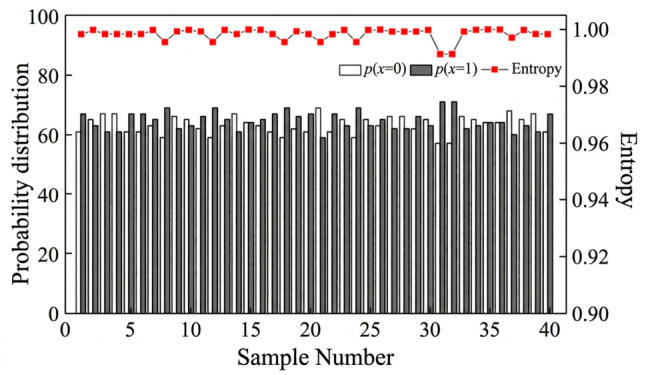
Information entropy of the PUF output response.

PUF randomness is commonly evaluated intuitively using information entropy. For the gas sensor PUF based on SnO_2_ nanoparticles, the information entropy of each output response group is greater than 0.98. The entropy of the gas sensor PUF based on Ni-SnO_2_ nanoparticles was calculated using Equation (3), and the results are shown in Figure 7. As can be seen, the information entropy of each output response group of the Ni-SnO_2_-nanoparticle-based gas sensor PUF exceeds 0.99, indicating that the GS-PUF based on Ni-SnO_2_ nanoparticles produces responses with improved randomness.

(3) $$E=-\sum_{r=0}^{1}p(r)\log_{2}p(r)$$

**Figure 8:**
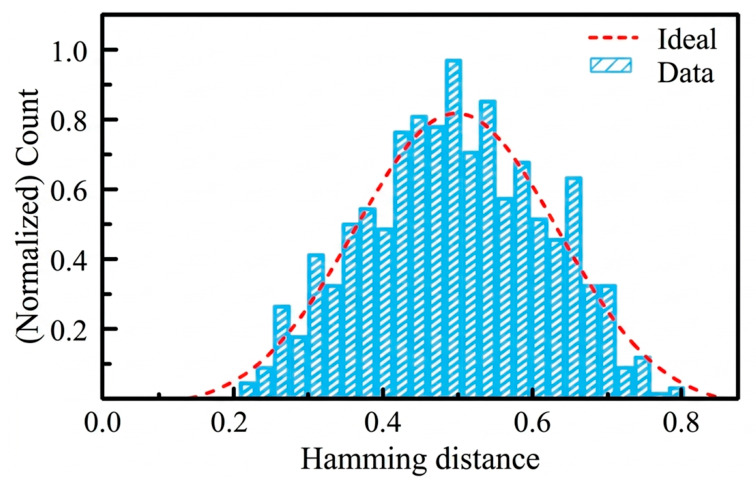
Uniqueness of the PUF output response.

Uniqueness reflects the distinguishability of responses from different devices under the same challenge, and it is evaluated using the inter-chip Hamming Distance (HD). Ideally, the uniqueness approaches 50%. In Equation (4), *k* denotes the number of PUF instances, $R_{i}$ and $R_{j}$ represent the output responses of the *i*-th and *j*-th PUF instances, respectively, and $HD(R_{i},R_{j})$ denotes the Hamming distance between the output responses. The uniqueness of the gas sensor PUF based on Ni-SnO_2_ nanoparticles is calculated to be 49.8%. The uniqueness of the sensing-type gas sensor PUF based on SnO_2_ nanoparticles was 45.16%. Therefore, the uniqueness is improved and approaches the ideal value of 50%, as shown in Figure 8.

(4) $$Uniqueness=\frac{2}{k(k-1)}\sum_{i=1}^{k-1}\sum_{j=i+1}^{k}\frac{HD(R_{i},R_{j})}{n}\times 100\%$$

To further clarify the effect of Ni doping on the sensing response and PUF metrics, the pristine SnO_2_-based gas sensor PUF and the Ni-SnO_2_-based GS-PUF are compared in Table 2.

As summarized in Table 2, the adopted Ni doping condition improves both the gas-sensing response and the statistical performance of the generated PUF responses. Compared with the pristine SnO_2_-based gas sensor PUF, the Ni-SnO_2_-based GS-PUF reduces the stabilization time from 48 s to 41 s. Meanwhile, the information entropy increases from greater than 0.98 to greater than 0.99, and the uniqueness improves from 45.16% to 49.8%, approaching the ideal value of 50%. These results indicate that Ni doping improves the sensing characteristics of SnO_2_ and helps extract more distinguishable device-to-device resistance features for high-quality PUF response generation.

**Figure 9:**
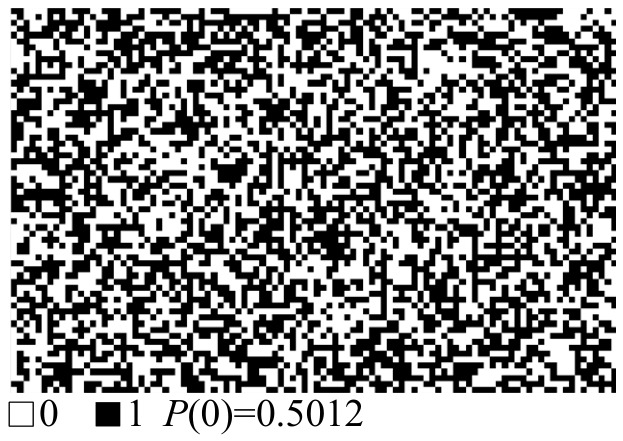
Two-dimensional mapping of the PUF output response.

Randomness characterizes the distribution of “0” and “1” in PUF responses. The PUF responses extracted from the electrical characteristics of the material were converted into a two-dimensional grayscale map, as shown in Figure 9. In the map, black squares represent logic “1” in the PUF response, whereas white squares represent logic “0”. From the grayscale map, the probability of logic “1” in the PUF response is calculated to be 0.4988, while that of logic “0” is 0.5012. The two values are uniformly distributed and close to the ideal value of 0.5, indicating that the PUF response exhibits good randomness.

The NIST randomness tests are used to analyze the statistical randomness of the output bitstream. In the NIST tests, the *p*-value quantifies the level of randomness. When the *p*-value is greater than 0.01, the bitstream is considered to achieve a 99% confidence level of randomness; generally, a larger *p*-value indicates a higher confidence in data randomness. Different NIST tests were performed on the PUF, and the results are listed in Table 3. As shown in the table, all listed NIST test items have passed. Moreover, the obtained PUF responses consistently exhibit relatively high *p*-values across the NIST test items, with the minimum *p*-value being 0.036815, and all *p*-values exceeding 0.01. These results confirm that the generated PUF responses possess good randomness.

**Figure 10:**
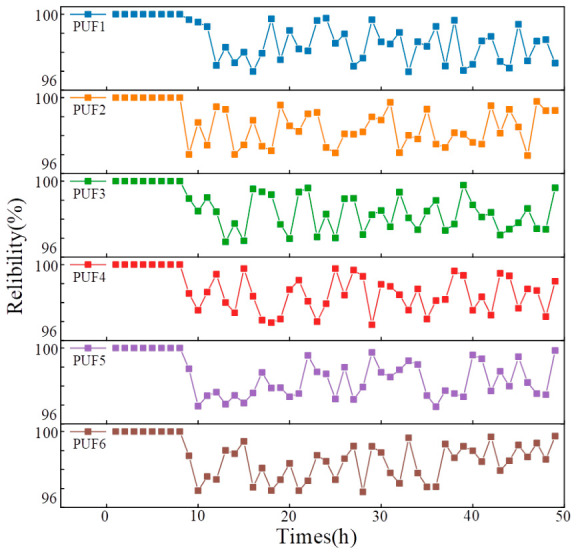
Reliability of the PUF output response.

Reliability also reflects the variation in responses over a given period. Six PUF samples were selected for continuous testing under fixed laboratory conditions. The first response of each PUF was used as the reference response, and the statistical results are shown in Figure 10. It can be observed that the reliability of all six PUF samples remains above 96%, indicating that the proposed Ni-SnO_2_-based GS-PUF can maintain stable response regeneration under the tested conditions.

Regarding power consumption, the proposed GS-PUF adopts an indirectly heated semiconductor gas-sensor structure. In the experimental setup, the heating wire of the gas-sensor unit was biased using a 4.5 V DC supply during response acquisition. Therefore, the main power consumption of the system originates from the heating elements required for gas sensing, rather than from the PUF response-extraction algorithm. The proposed design should be understood as a low-additional-security-circuit-overhead approach, because it reuses the intrinsic response variations of the existing gas-sensor array and does not require a dedicated silicon PUF circuit. In the present work, the heating current of each sensor and the total array-level power consumption were not separately measured. Therefore, quantitative power characterization and system-level power optimization of the indirectly heated GS-PUF remain important topics for future work.

Since metal-oxide gas sensors are sensitive to environmental conditions, ambient humidity is an important factor for the proposed GS-PUF. Water molecules may affect the adsorption/desorption behavior of oxygen species and ethanol molecules on the Ni-SnO_2_ surface, thereby influencing the baseline resistance, response amplitude, and response/recovery dynamics of the sensors. These humidity-induced resistance variations may further affect the intra-device Hamming distance, reliability, information entropy, and bit uniformity of the generated PUF responses. In the current work, the long-term reliability test was conducted under fixed laboratory conditions, and systematic temperature-/humidity-dependent long-term measurements were not performed. Therefore, the influence of temperature and humidity on entropy and reliability is considered a limitation of the present study. In future work, environmental robustness tests under controlled temperature and relative humidity conditions will be carried out, and compensation strategies such as adaptive feature selection, calibration, and error-correction mechanisms will be incorporated to improve the stability of GS-PUF responses under non-ideal conditions.

The comparison between this work and recent sensor-based and non-sensor PUFs is presented in Table 4. As shown, the proposed Ni-SnO_2_ gas-sensor-based PUF achieves a uniqueness of 49.8%, which is close to the ideal value of 50%, and its reliability remains above 96% under fixed laboratory conditions. Compared with non-sensor PUFs based on subthreshold circuits, the proposed design reuses the intrinsic response variations of gas sensors as entropy sources and does not require an additional dedicated silicon PUF circuit. In addition, the proposed GS-PUF generates a 128-bit response and exhibits an information entropy greater than 0.99, indicating good randomness and low output bias.

Overall, the comparison shows that the proposed GS-PUF achieves competitive uniqueness and reliability while directly utilizing gas-sensor response variations. This feature makes the proposed design suitable for lightweight sensing-security integration in IoT nodes. However, quantitative modeling-attack evaluation and environmental robustness tests will still be required in future work.

## 5. Conclusions

Focusing on the security requirements of the IoT perception layer, this paper proposes a GS-PUF design based on a Ni-doped SnO_2_ nanoscale gas sensor array. By introducing a material-level randomness enhancement mechanism and a circuit/algorithm-level random resistance balancing strategy, stable extraction of high-quality multi-bit PUF responses is achieved. Experimental results show that the constructed PUF approaches ideal performance in terms of randomness, uniformity, and uniqueness, with an information entropy greater than 0.99, an output bit distribution close to 0.5, and a uniqueness of 49.8%. The PUF also passes all NIST randomness tests, and its long-term reliability remains above 96% under fixed laboratory conditions, verifying the feasibility of the proposed method for lightweight hardware security. In addition, compared with conventional silicon-based PUF structures, the proposed scheme fully exploits the intrinsic characteristics of sensors and requires no additional complex circuitry, thereby providing a low-cost implementation pathway for sensing-security integration.

Future work can be further advanced in the following directions. First, at the device level, various functional materials, such as multi-doped materials or heterojunction structures, can be explored to further enhance the complexity of entropy sources and environmental robustness. Second, at the algorithm and architecture levels, adaptive feature selection and error-correction mechanisms can be incorporated to improve stability under non-ideal conditions such as temperature, humidity, and voltage fluctuations. In addition, systematic temperature- and humidity-dependent reliability tests will be conducted to evaluate the environmental robustness of the proposed GS-PUF, and compensation mechanisms will be explored to maintain stable entropy and reliability under practical operating conditions. Third, from a security perspective, systematic machine-learning-based modeling attack evaluations, such as logistic regression, support vector machine, random forest, and multilayer perceptron analyses, will be carried out in future work to quantitatively assess the modeling resistance of the proposed GS-PUF. Finally, at the system application level, this type of sensor PUF can be integrated with edge computing, trusted execution environments, and related technologies to construct an end-to-end lightweight security system for IoT applications.

## Figures and Tables

**Table 1:** Pseudocode of the 8-bit random resistance balancing algorithm.

Pseudocode of the 8-bit random resistance balancing algorithm
1: **int** *bit*[*place*]; 2: **int** *left_idx* [3]; 3: **int** *right_idx* [3]; 4: **double** *v* [8]; 5: *i* = 0; 6: **do** {*lsum* = *v*[(*i* + *left_idx* [0]) mod 8] + *v*[(*i* + *left_idx* [1]) mod 8] + *v*[(*i* + *left_idx* [2]) mod 8] 7: *rsum* = *v*[(*i* + *right_idx* [0]) mod 8] + *v*[(*i* + *right_idx* [1]) mod 8] + *v*[(*i* + *right_idx* [2]) mod 8]}; 8: **if** *lsum* > *rsum*; 9: **then** *bits*[*place*] = 1; 10: **else** *bits*[*place*] = 0; 11: *place* = *place* + 1; 12: **while** (*i* < 8); 13: **return**

**Table 2:** Effect of Ni doping on sensing response and PUF metrics.

Material	Ni Dopant Source	Stabilization Time	Information Entropy	Uniqueness
SnO_2_	-	48 s	>0.98	45.16%
Ni-SnO_2_	NiCl_2_·2H_2_O, 17 mg	41 s	>0.99	49.8%

**Table 3:** NIST randomness test.

Test Name	*P*_1_-Value	Pass
Approximate Entropy	0.036815	Yes
Block Frequency	0.182565	Yes
Cumulative Sums	0.629223	Yes
FFT	0.908677	Yes
Frequency	0.841481	Yes
Linear Complexity	0.978469	Yes
Longest Run	0.837344	Yes
Runs	0.599929	Yes
Overlapping Template	0.978025	Yes
Rank	0.106919	Yes
Serial	0.355363	Yes

**Table 4:** Comparison with recent sensor-based and non-sensor PUFs.

	Method	Category	Uniqueness	Reliability	Key
[29]	Piezoelectric-based	Sensor-based	47.24%	96.07%	128-bit
[28]	Gyroscopes-based	Sensor-based	About 50%	-	128-bit
[32]	Accelerometer-based	Sensor-based	42.64%	92.17%	**-**
[33]	Subthreshold current array	Non-sensor	52.8%	91%	65-bit
[34]	Subthreshold divider	Non-sensor	50.29%	89.1%	600-bit
**This** **work**	**Gas sensors-based**	Sensor-based	**49.8%**	**>96%**	**128-bit**

## Data Availability

The original contributions presented in this study are included in the article. Further inquiries can be directed to the corresponding authors.
